# Prognostic significance and immune landscape of a cell cycle progression-related risk model in bladder cancer

**DOI:** 10.1007/s12672-024-01008-x

**Published:** 2024-05-12

**Authors:** Zhouting Tuo, Yuan Lin, Ying Zhang, Liang Gao, Dexin Yu, Jiani Wang, Chenyu Sun, Xianchao Sun, Jinyou Wang, Apurwa Prasad, Nimarta Bheesham, Muzi Meng, Zhengmei Lv, Xin Chen

**Affiliations:** 1 Department of Urology, The Second Affiliated Hospital of Anhui Medical University, Hefei, Anhui China; 2 Center for Clinical Medicine, Huatuo Institute of Medical Innovation (HTIMI), Berlin, Germany; 3 Institute for Social Medicine, Charité-Universitätsmedizin Berlin, Corporate Member of Freie Universität Berlin, Humboldt-Universität Zu Berlin; Berlin Institute of Health, Epidemiology and Health Economics, Berlin, Germany; 4 Department of General Surgery, The Second Affiliated Hospital of Anhui Medical University, Hefei, Anhui China; 5 Parkview Regional Medical Center, 11109 Parkview Plaza Dr, Fort Wayne, IN 46845 USA; 6 Internal Medicine, University of Illinois College of Medicine, One Illini Drive, Peoria, IL 61605 USA; 7 UK Program Site, American University of the Caribbean School of Medicine, Vernon Building Room 64, Sizer St, Preston, PR1 1JQ UK; 8 Bronxcare Health System, 1650 Grand Concourse, The Bronx, NY 10457 USA; 9 Department of Histology and Embryology, School of Basic Medical Sciences, Anhui Medical University, Anhui, China

**Keywords:** Cell cycle progression, Bladder cancer, Prognosis, Immune landscape, Nomogram

## Abstract

**Background:**

A greater emphasis has been placed on the part of cell cycle progression (CCP) in cancer in recent years. Nevertheless, the precise connection between CCP-related genes and bladder cancer (BCa) has remained elusive. This study endeavors to establish and validate a reliable risk model incorporating CCP-related factors, aiming to predict both the prognosis and immune landscape of BCa.

**Methods:**

Clinical information and RNA sequencing data were collected from the GEO and TCGA databases. Univariate and multivariate Cox regression analyses were conducted to construct a risk model associated with CCP. The performance of the model was assessed using ROC and Kaplan–Meier survival analyses. Functional enrichment analysis was employed to investigate potential cellular functions and signaling pathways. The immune landscape was characterized using CIBERSORT algorithms. Integration of the risk model with various clinical variables led to the development of a nomogram.

**Results:**

To build the risk model, three CCP-related genes (RAD54B, KPNA2, and TPM1) were carefully chosen. ROC and Kaplan–Meier survival analysis confirm that our model has good performance. About immunological infiltration, the high-risk group showed decreased levels of regulatory T cells and dendritic cells coupled with increased levels of activated CD4 + memory T cells, M2 macrophages, and neutrophils. Furthermore, the nomogram showed impressive predictive power for OS at 1, 3, and 5 years.

**Conclusion:**

This study provides new insights into the association between the CCP-related risk model and the prognosis of BCa, as well as its impact on the immune landscape.

**Supplementary Information:**

The online version contains supplementary material available at 10.1007/s12672-024-01008-x.

## Introduction

One common cancer of the urinary tract that has a high morbidity and mortality rate is bladder cancer (BCa) [1]. It ranks tenth among common malignancies, encompassing non-muscle-invasive and muscle-invasive tumors [2, 3]. Although many possible biomarkers for BCa diagnosis and treatment have been identified, their effectiveness differs throughout patients despite breakthroughs in bioinformatics and sequencing [4, 5]. Therapy choices are still guided by radiographic and pathologic examinations [6]. BCa progression is influenced by multiple biological pathways, with cell cycle progression (CCP) playing a pivotal role [7]. Bladder cancer cells acquire resistance through a myriad of mechanisms intertwined with the cell cycle, enabling them to persistently divide even when subjected to drug treatments [8, 9].

For cells to function properly, four phases of the carefully controlled cell cycle are required [10]. Inappropriate cell cycle progression can lead to unchecked cell proliferation and the advancement of cancer, particularly at critical checkpoints like G1/S and G2/M [11]. Dysregulation of CCP is implicated in various diseases, including cancer [12–14]. Aberrations in genes governing CCP and apoptotic pathways contribute to tumorigenesis and progression [15, 16]. Clinical and basic research has revealed CCP-related genes as prognostic indicators across cancer types [17–19]. Previous studies have described that the CCP-related ANLN is abnormally expressed in kidney cancer and promotes carcinogenesis by activating PI3K/Akt/mTOR signaling [20]. CCP-related genes exhibit abnormal expression patterns across a spectrum of human tumors, presenting significant potential as prognostic markers.[21–23]. These CCP-related genes haven’t been fully examined, yet, and further research is needed to fully understand the immunological and clinical significance of bladder cancer.

Even with this understanding, it is still unclear exactly what functions, prognosis, and immunological environment CCP-related gene markers play in BCa. Through thorough bioinformatics research, a CCP-related risk model is to be built in our study to systematically predict the immunological landscape and prognosis of BCa.

## Materials and methods

### Data collection

The databases Gene Expression Omnibus (GEO) and The Cancer Genome Atlas (TCGA) were used to get clinical and RNA sequencing data. There were 165 BCa samples in the GSE13507 dataset, compared to 433 samples in the TCGA-BLCA cohort, which included 414 tumor and 19 normal samples [24].The Cancer Cell Line Encyclopedia (CCLE) database served as the source of sequencing information for cancer cell lines [25]. The source of CCP-related genes was the Molecular Signature Database (MSigDB) [26]. Using the “limma” package with a threshold of |log2FC|> 1 and a false discovery rate < 0.05, differentially expressed genes (DEGs) were found.

### Identification and validation of the CCP-related risk model

The “survival” and “survminer’’ packages were used to identify prognostic CCP-related genes; previous literature techniques are cited [27]. In order to identify important genes and create risk models, univariate and multivariate Cox regression analyses were carried out. The analysis of the least absolute shrinkage and selection operator (LASSO) was made easier by the "glmnet" and "survival" programs. The LASSO regression coefficients and gene expression levels were used to calculate risk scores, which were calculated using the following formula: risk score = EXP(gene1) * coefficient(gene1) + EXP(gene2) * coefficient(gene2) + … + EXP(genex) * coefficient(genex). Based on the median risk score, patients were then divided into high-risk and low-risk groups. Using the "survival" package and the TCGA-BLCA cohort as well as the GSE13507 dataset, Kaplan–Meier (KM) survival analysis was used to confirm the gene signature's predictive efficacy for overall survival (OS) in BCa. The “survivalROC” program was used to perform receiver operating characteristic (ROC) analysis. The R software's "maftools" package was utilized to profile somatic mutations in genes linked to CCP [28]. With reference to earlier literature studies, an RNA expression based (full set of accessible genes) score was computed to assess the cancer stemness index (RNA stemness score, RNAss) [29]. Referring to other research methods [27], the link between the RNA stemness score and the CCP-related model risk score was evaluated.

### Immune infiltration analysis

To calculate the percentages of immune cell infiltration in TCGA samples, CIBERSORT algorithms were utilized [30]. The investigation then went on to assess immune cell infiltration and other immunological functions among various risk categories.

### Functional enrichment analysis

By integrating the “clusterProfiler”, “enrichplot”, and “ggplot2’’ packages, functional enrichment analysis was carried out using Gene Ontology (GO) and Kyoto Encyclopedia of Genes and Genomes (KEGG) [31]. Furthermore, the “limma”, “GSEABase”, and “GSVA” packages were used to perform Gene Set Variation Analysis (GSVA) [32, 33].

### Independent prognosis analysis

Using the “survival” package, univariate and multivariate Cox regression analysis were carried out. The “survival”, “survminer”, and “timeROC’’ programs in R software were then used to assess the CCP-related risk model with a number of clinical factors, with reference to earlier literature methods [27].

### Statistical analysis

R software, a statistically sound program with extensive literature documentation, was used for data processing (version 4.2.1). Every statistical and data processing technique has reference to earlier publications in the literature [12, 22, 27]. The *t-test* was used to compare groups, and *P* < 0.05 was chosen as the threshold for statistical significance.

## Results

### Identification of prognostic CCP-related risk model in BCa

**Fig. 1:**
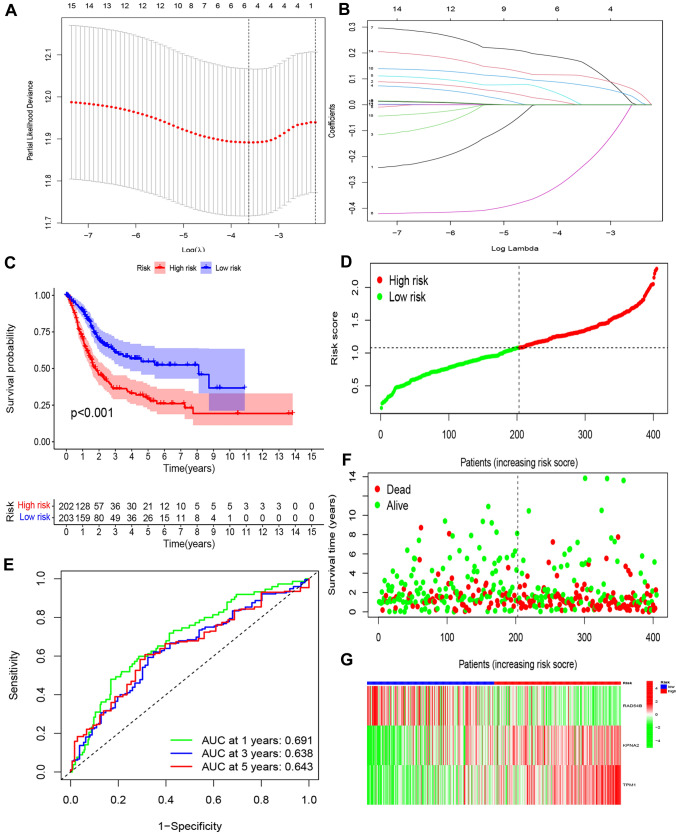
Generation and Validation of a CCP-related risk model in BCa. **A** Tenfold cross-validation for tuning parameter selection in the LASSO model. **B** LASSO coefficient profiles of CCP-related genes. **C** Kaplan–Meier curves of OS in BCa from TCGA database. **D** Distribution of risk scores among BCa patients. **E** Time-dependent ROC curves and AUCs of BCa patients for 1-year, 3-year, and 5-year survival predictions.** F** Distribution of patients status for each risk score among BCa patients. **G** Heatmap of three CCP-related genes among BCa patients

To identify differences in CCP-related genes, differential expression analysis was carried out using heat maps and volcano plots (Fig. S1A,B). Fifteen CCP-related genes, including ABL1, APBB1, and AURKA, were found to have prognostic significance through a univariate Cox regression analysis (Table 1). The predictive significance of these 15 genes was subsequently evaluated using Lasso regression analysis (Fig. 1A, B). Finally, multivariate Cox regression analysis led to the selection of three genes (RAD54B, KPNA2, and TPM1) for the construction of the CCP-related risk model (Table 2).

**Table 1:** Univariate Cox regression analysis of CCP-related genes in BLCA

Gene	HR	HR.95L	HR.95H	*P* value
ABL1	1.291616523	1.010733216	1.650557454	0.040825833
APBB1	1.164436026	1.004089317	1.35038909	0.044014624
AURKA	1.1962973	1.013092595	1.41263221	0.034571795
BUB1B	1.208760962	1.00168825	1.458640513	0.047978564
RAD51	1.269164851	1.004885223	1.602948658	0.045406135
RAD54B	0.677813405	0.500604289	0.917752848	0.011902004
KPNA2	1.297435549	1.073644439	1.567873817	0.007026833
LATS2	1.377515209	1.101761154	1.722286309	0.004949582
PAM	1.240411219	1.056654918	1.456123439	0.008448065
TPD52L1	1.262738908	1.086998672	1.466891902	0.002281042
SPP1	1.079076368	1.003253803	1.160629349	0.040623319
TAGLN	1.124597689	1.02406645	1.234997946	0.013982678
THBS1	1.180510737	1.052578972	1.323991489	0.00457444
TPM1	1.254824189	1.098445403	1.433465642	0.000829877
TPM2	1.127667185	1.012437035	1.256012213	0.028909936

**Table 2:** Multivariate Cox regression analysis of three CCP-related genes in BLCA

Gene	coefficient	HR	HR.95L	HR.95H	*P* value
RAD54B	−0.457119469	0.633104699	0.454142742	0.882589377	0.007001225
KPNA2	0.319838225	1.376904998	1.118383957	1.6951847	0.00257421
TPM1	0.170742733	1.186185544	1.028450023	1.36811329	0.019012135

### Validation of the CCP-related risk model

A risk score was computed using the mRNA expression data and coefficient values of these three risk genes. On the basis of the median risk score threshold, patients were subsequently divided into high-risk and low-risk groups. Using Kaplan–Meier survival analysis, the CCP-related risk model's ability to predict OS in BCa patients was evaluated. The results of the research showed that BCa patients in the low-risk group had survival outcomes that were noticeably better than those in the high-risk group (Fig. 1C, D). The model's strong predictive accuracy was indicated by ROC analysis, which showed area under the curve (AUC) values of 0.691, 0.638, and 0.643 for 1-year, 3-year, and 5-year survival, respectively (Fig. 1E). Furthermore, the model's heatmap of risk scores and survival results matched the classification of high-risk and low-risk groups quite well (Fig. 1F, G). Moreover, the GEO database was used to validate the risk model (Fig. S2A-E).

### Mutation profiles and RNA stemness scores analyses

**Fig. 2:**
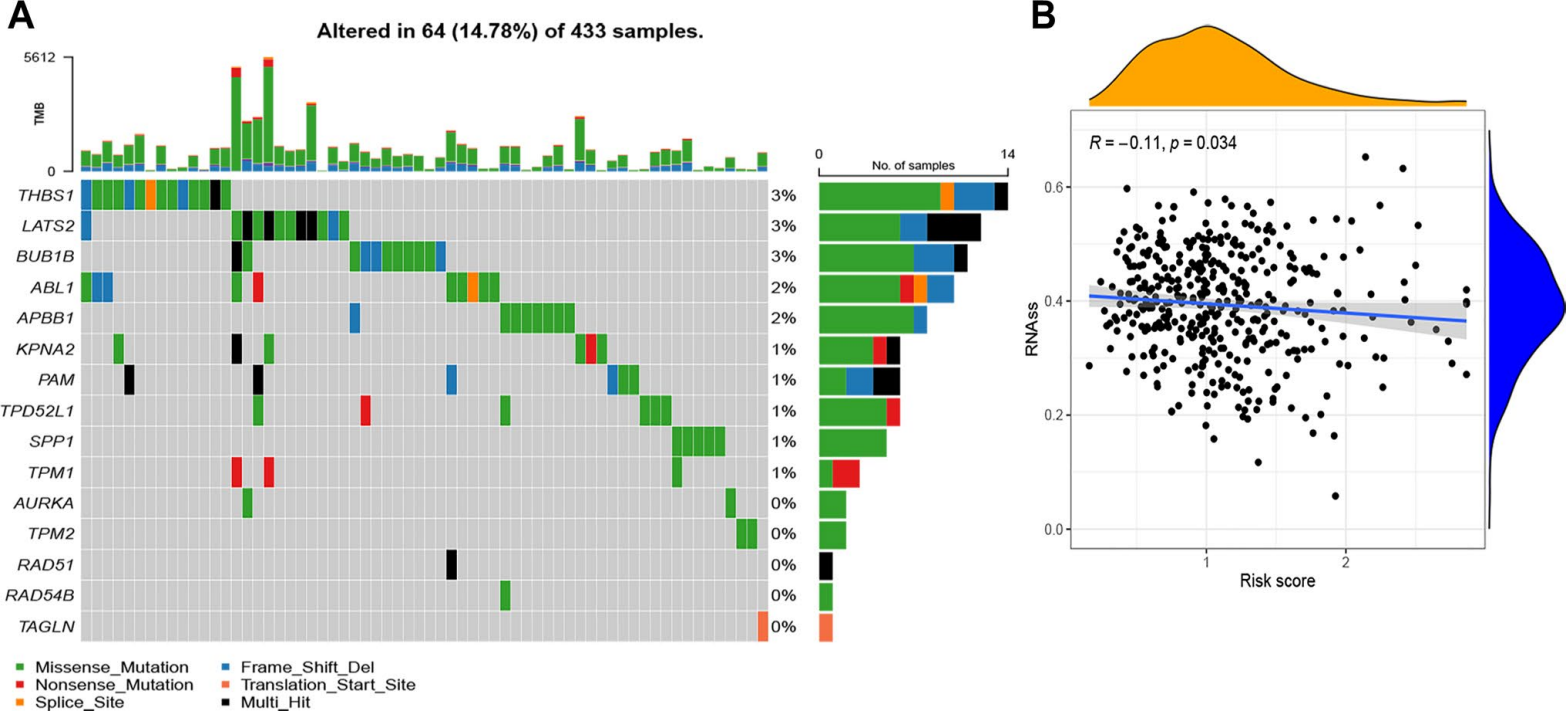
Mutation profiles and stemness association of the CCP-related risk model. **A** The mutation profiles of CCP-related 15 genes. **B** The association between the RNA stemness score and the CCP-related model risk score

The gene mutation rate for the three CCP-related risk model genes was significantly lower at just 2%, while the mutation rates among the 15 CCP-related risk genes in BCa patients were determined to be 14.78% (Fig. 2A). Additionally, a little negative correlation (R = −0.11, p = 0.034) was found between the risk score obtained from the CCP-related risk model and the RNAss (Fig. 2B).

### Immune infiltration cells of the CCP-related risk model

**Fig. 3:**
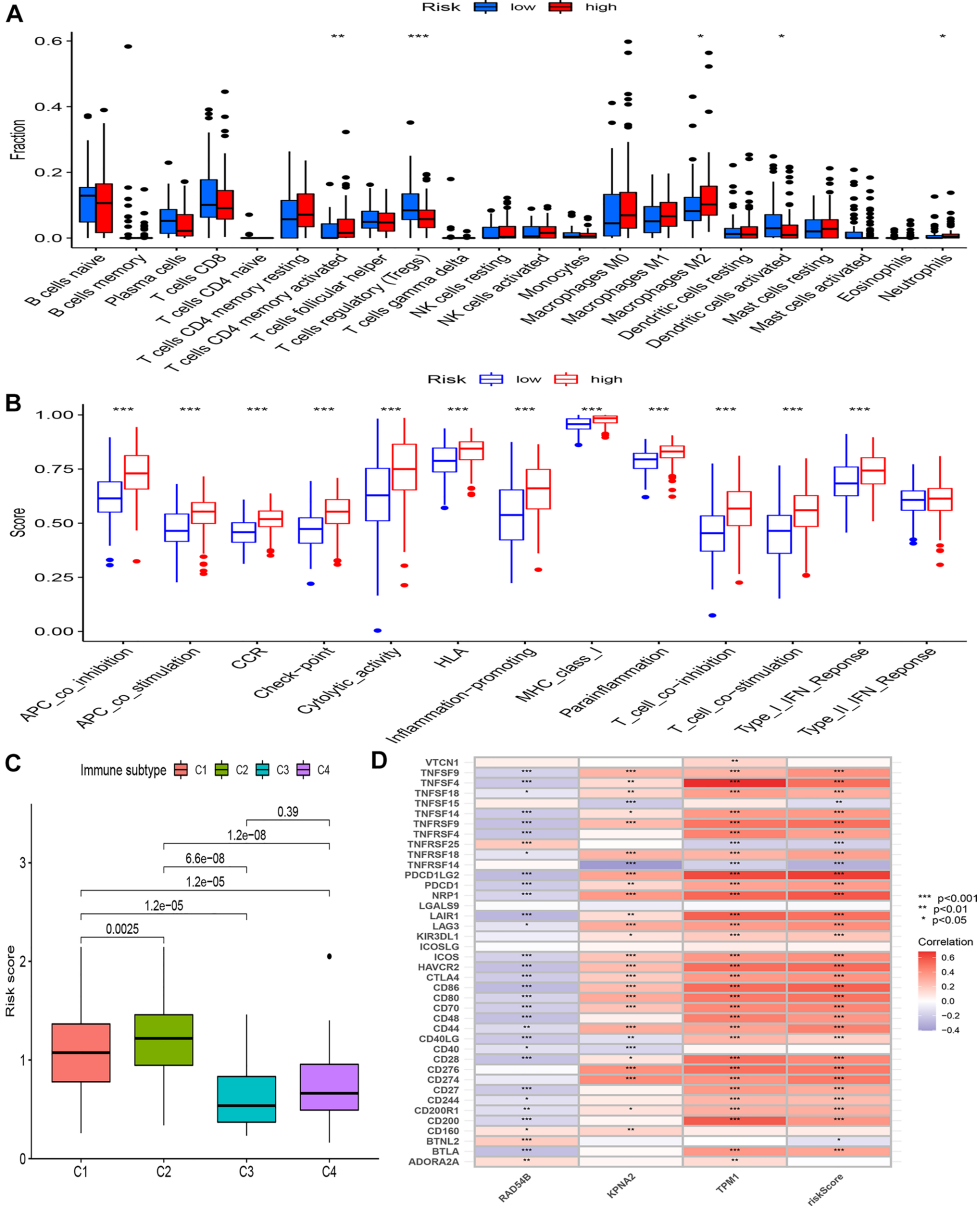
Association between CCP-related risk model and immune landscape in BCa.** A** Diverse immune cells infiltration between high-risk and low-risk groups. **B** Diverse immune-related function between high-risk and low-risk groups.** C** Association between CCP-related risk model and immune subtypes. **D** Correlation between the expression of immune checkpoints and the CCP-related genes and risk scores. *p < 0.05, **p < 0.01, ***p < 0.001, ****p < 0.0001, ns: not significant

The immune landscape and the CCP-related risk model were correlated, and the results showed that the high-risk group had lower infiltration of regulatory T cells and dendritic cells and increased infiltration of activated CD4 + memory T cells, M2 macrophages, and neutrophils (Fig. 3A). Furthermore, the group at high risk demonstrated increased levels of multiple immune functions, such as co-inhibition of antigen-presenting cells (APCs), co-stimulation of APCs, CCR, checkpoint, cytolytic activity, HLA, promotion of inflammation, MHC class I, parainflammation, co-inhibition of T cells, co-stimulation of T cells, and Type I IFN response (Fig. 3B). Additionally, we examined the CCP-related risk model's risk score for each of the four immunological subtypes. Notably, immune cells of the C2 (IFN‐γ dominant type) had a risk score that was considerably greater than those of the C1 (immunodepressive type), C3 (inflammatory type), and C4 (immunologically balanced type) (Fig. 3C). Additionally, we looked into the relationship between the risk score, three genes connected to CCP, and 40 immune-associated genes (Fig. 3D). Additionally, the relationship between the risk score and other immune cell groups was investigated using a variety of calculating algorithms, including XCELL, TIMER, CIBERSORT-ABS, and CIBERSORT. Stronger correlations with the risk score were seen with Th2 CD4 + T cells, myeloid dendritic cells, macrophages M1, cancer-associated fibroblasts, and macrophages M2 (Fig. S3).

### Functional enrichment analyses

**Fig. 4:**
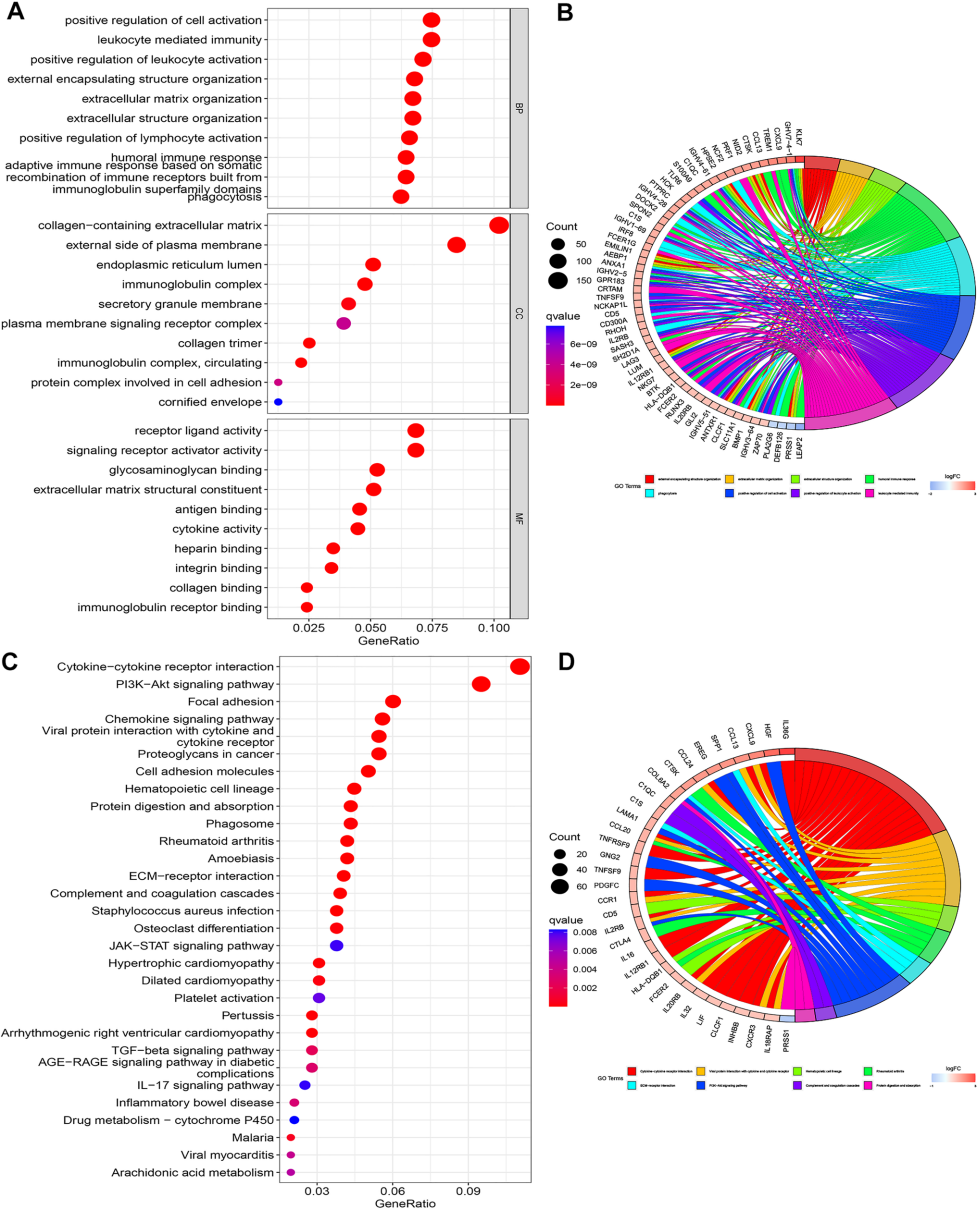
Functional enrichment analysis of the CCP-related risk model. **A** Go enrichment analysis of the CCP-related risk model.** B** Circos profile of the different genes enriched in various GO terms.** C** KEGG enrichment analysis of the CCP-related risk model.** D** Circos profile of the different genes enriched in various KEGG terms

We investigated the biological roles and underlying processes of the CCP-related risk model using GO and KEGG enrichment analysis. Numerous important biological processes (BP) were identified by GO enrichment analysis, including “leukocyte-mediated immunity,” “positive regulation of leukocyte activation,” and “positive regulation of cell activation” (Fig. 4A). “Collagen-containing extracellular matrix,” “external side of the plasma membrane,” and “endoplasmic reticulum lumen” were the three most common enriched cellular components (CC) (Fig. 4A). Moreover, “receptor ligand activity,” “signaling receptor activator activity,” and “glycosaminoglycan binding” were shown to be enriched molecular functions (MF) (Fig. 4A). Several GO keywords were enriched in differentially expressed genes, including KLK7, GHV7-4–1, and CXCL9 (Fig. 4B). Potential connections between the CCP-related risk model and biological pathways like “cytokine-cytokine receptor interaction,” “PI3k-akt signaling pathway,” and “focal adhesion” were shown by KEGG enrichment analysis (Fig. 4C). Furthermore, certain KEGG keywords were enriched in differentially expressed genes, such as IL36G, HGF, and CXCL9 (Fig. 4D).

**Fig. 5:**
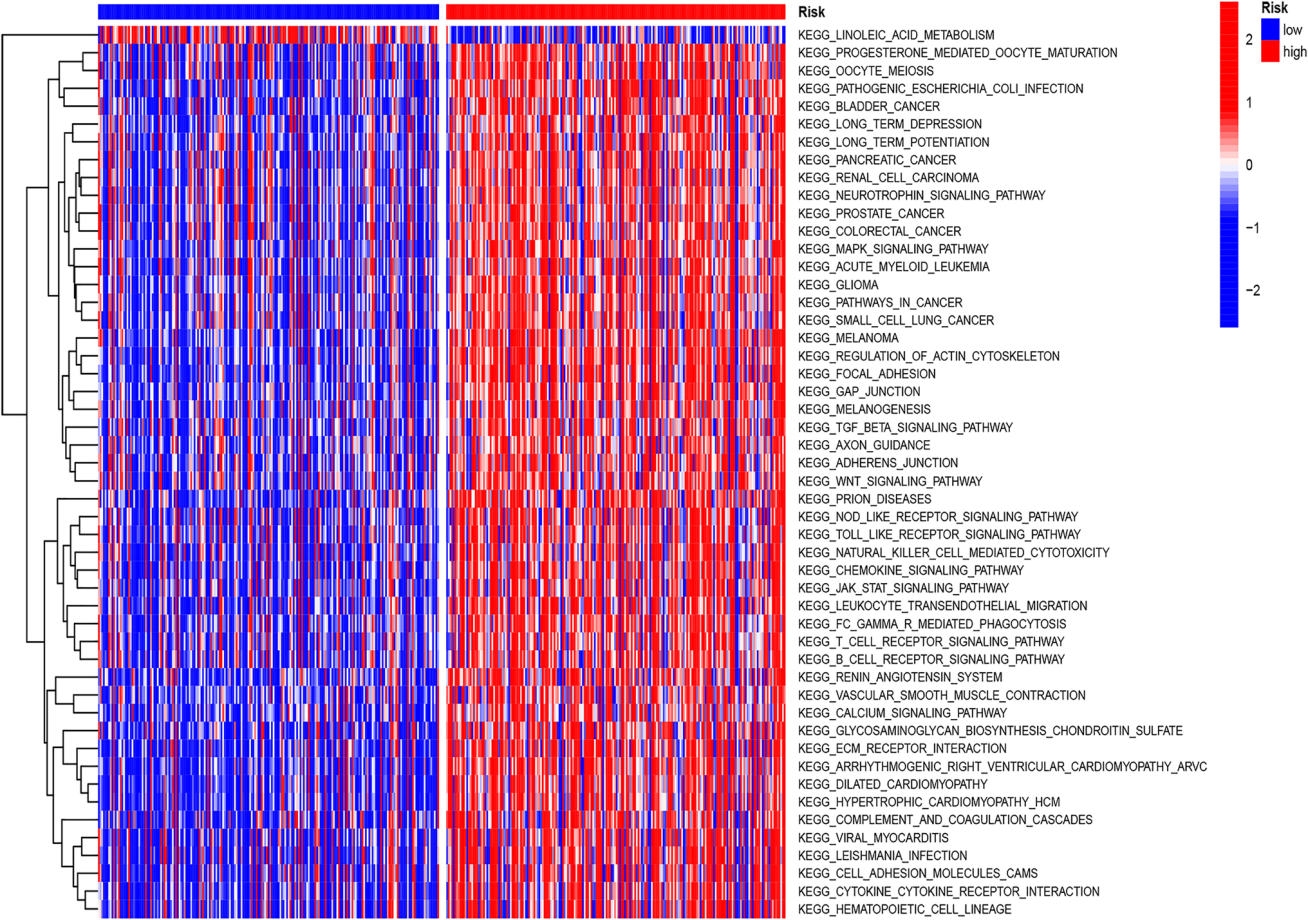
GSVA of the potential pathways enriched in high-risk and low-risk groups

To evaluate potential pathways, GSVA was used. The results showed that the risk associated with the CCP-related risk model was positively correlated with the scores of the “linoleic acid metabolism,” “progesterone-mediated oocyte maturation,” and “oocyte meiosis” pathways (Fig. 5). Remarkably, the high-risk group also exhibited an enhanced bladder cancer pathway (Fig. 5).

### Independent prognostic value of the CCP-related risk model

**Fig. 6:**
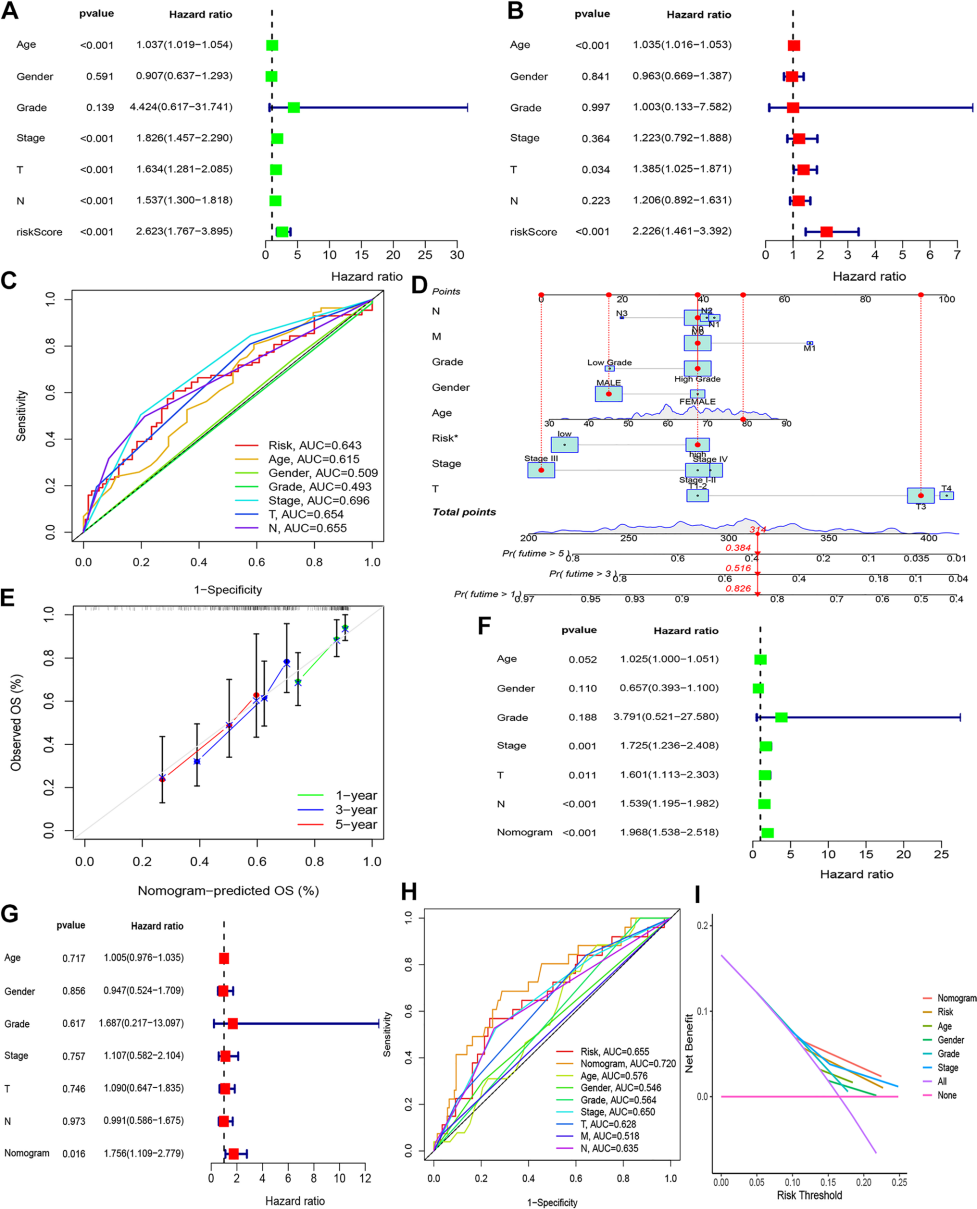
Prognostic analysis of CCP-related risk scores and clinical parameters in the BCa cohort. **A** Forest plot of a univariate Cox regression analysis of CCP-related risk scores and clinical parameters. **B** Forest plot of a multivariate Cox regression analysis of CCP-related risk scores and clinical parameters. **C** Time-dependent multivariate ROC curves and AUCs of CCP-related risk scores and clinical parameters for OS predictions. **D** The construction of a nomogram with CCP-related risk scores and clinical parameters. **E** The predicted OS at 1, 3, and 5 years with the nomogram. **F** Forest plot of a univariate Cox regression analysis of the nomogram and clinical parameters. **G** Forest plot of a multivariate Cox regression analysis of the nomogram and clinical parameters. **H** Time-dependent multivariate ROC curves and AUCs of nomogram and clinical parameters for OS predictions. **I** Decision curve analysis for the nomogram, risk, and clinical variables

The risk model's prognostic value was assessed using univariate and multivariate Cox regression analysis. Age, clinical stage, T stage, N stage, and the risk score of the CCP-related risk model were found to be prognostic factors for BCa patients in the univariate analysis (Fig. 6A). But in the multivariate analysis, the risk score of the CCP-related risk model, T stage, and age were the only variables that remained independent prognostic predictors (Fig. 6B). Additionally, multivariate ROC curve analysis produced an AUC of 0.696 for the clinical stage and 0.643 for the CCP-related risk model (Fig. 6C).

### Establishment of a nomogram and risk model comparison

A predictive nomogram was created by combining different clinicopathological characteristics with the CCP-related risk model gene features. Based on the research, the nomogram’s only predictive element was the CCP-related risk model (Fig. 6D). We constructed predictive models by integrating numerous clinical data of these individuals to validate the performance of our nomogram. High agreement with reported OS rates was found when OS at 1, 3, and 5 years was assessed using the nomogram (Fig. 6E). Furthermore, the strong predictive potential of the nomogram was validated by univariate Cox regression analysis (HR = 1.968, 95% CI 1.538—2.518, *p* < 0.001) (Fig. 6F). Nonetheless, the multivariate analysis verified the nomogram's prognostic potential (HR = 1.756, 95% CI 1.109—2.779, *p* < 0.05) (Fig. 6G). Moreover, multivariate ROC curve analysis revealed that the nomogram’s and the risk model’s respective AUCs were 0.720 and 0.655 (Fig. 6H). Nomogram demonstrated a higher overall net benefit in patients with bladder cancer, according to decision curve analysis (Fig. 6I).

### Expression and prognosis analysis of three CCP-related risk genes

**Fig. 7:**
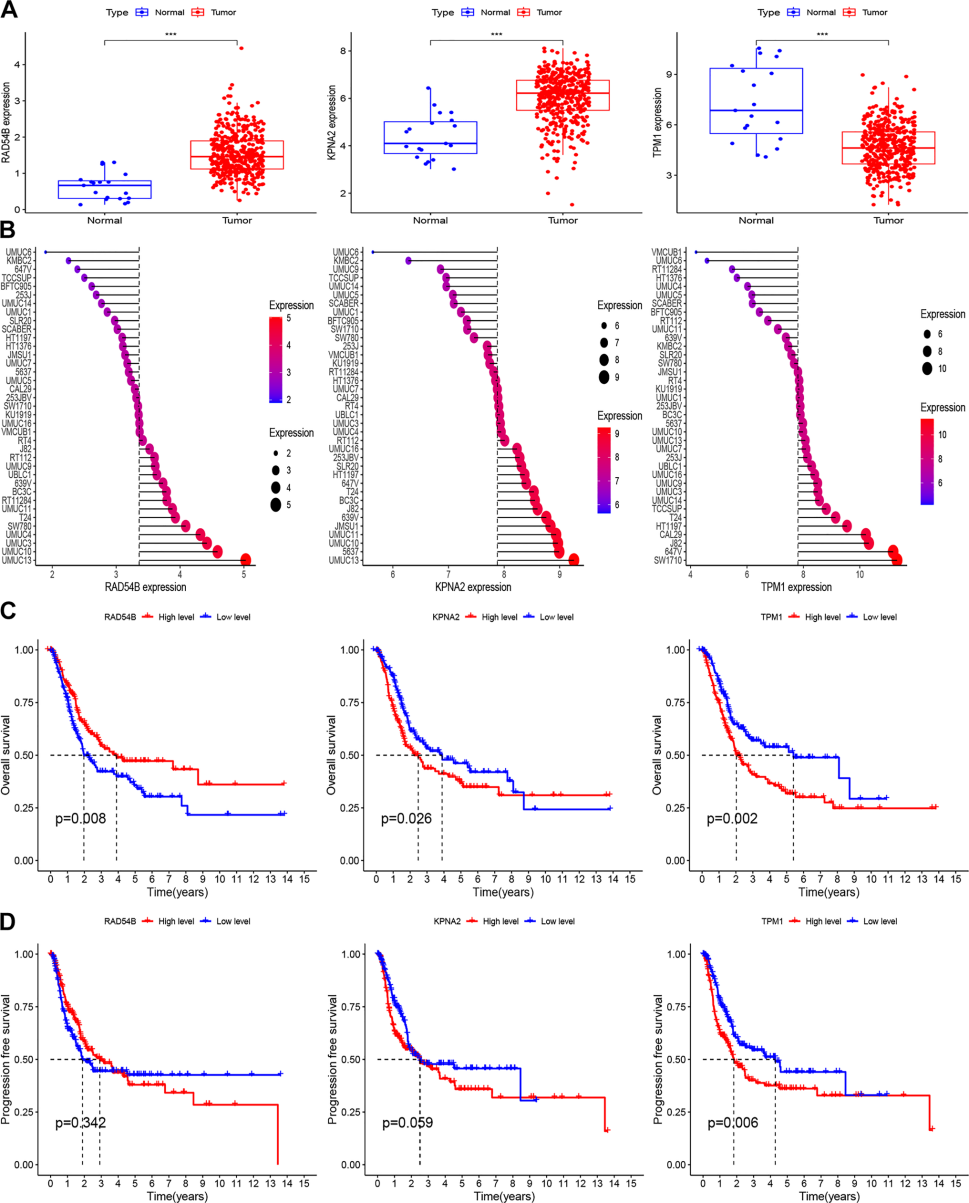
Expression and prognosis analysis of three CCP-related risk genes. **A** The mRNA expression of three CCP-related risk genes in TCGA-BLCA cohort. **B** The expression level of three CCP-related risk genes in bladder cancer cell lines based on CCLE database. **C** KM survival curves of OS probability based on the expression profile of three CCP-related genes (i.e. RAD54B、KPNA2 and TPM1). **D** KM survival curves of PFS probability based on the expression profile of three CCP-related genes (i.e. RAD54B、KPNA2 and TPM1)

Drawing from the TCGA-BLCA cohort, we discovered that RAD54B and KPNA2 expressions rose in tumor tissues, whereas TPM1 expressions declined in comparison to corresponding normal tissues (Fig. 7A). Three risk genes' expressions were examined after we extracted the BCa cell line expression data from the CCLE database (Fig. 7B). KM survival curves predicting the survival outcomes associated with three CCP-related genes in BCa. Low expression of RAD43B and high expression of KPNA2 and TPM1 were notably linked to poor overall survival in BCa patients (Fig. 7C). Furthermore, elevated levels of TPM1 were indicative of worse progression-free survival (Fig. 7D).

## Discussion

Because of its high mortality, morbidity, and burden of medical treatment, BCa is a serious health concern, having more than doubled in occurrence worldwide in the last two decades [34, 35]. A concentrated effort has been made over the last thirty years to investigate novel therapeutic, prognostic, and diagnostic biomarkers for BCa patients in an attempt to determine which patients could benefit the most from treatment. Individual or combined genomes that identify unique patterns of gene expression inside disease processes have been found to be useful for prognostic prediction and disease classification [36, 37]. Anomalies in a number of cellular pathways, most notably the disruption of cell cycle control, are thought to play a role in the carcinogenesis and development of BCa [38, 39]. According to earlier studies, CCP-related gene malfunctions can influence immune cell infiltration during the initiation and spread of cancer as well as cause unchecked cell proliferation [38, 40]. Aberrations in CCP-related genes have been associated with unfavorable prognostic outcomes across various human neoplastic diseases [41, 42]. Three selected genes (RAD43B, KPNA2, and TPM1) were used to create a risk model, and CCP-related genes were evaluated using various bioinformatics databases and techniques to evaluate their biological function, predictive power, and relationship to the immune microenvironment in BCa.

The cell cycle process is intimately associated with the three genes connected to CCP. According to earlier studies, homologous recombination repair is inhibited by RAD54B knockdown, and ovarian tumor tissues with RAD54B mutations have been shown to have more DNA double-strand breaks than normal tissues [43]. KPNA2 normally expresses itself at a low level in normal tissues, but in some carcinomas, it has been shown to be overexpressed, which can affect the immune system, tumor cell proliferation, and differentiation [44–46]. There has been discussion on the physiological function of TPM1 in various malignancies; tumor types have been linked to metastasis and cancer progression at both higher and lower expression levels [47, 48]. The results of our investigation show that, in BCa, the CCP-related risk model has better predictive efficacy than the TNM stage. Furthermore, using both univariate and multivariate Cox regression analysis, the CCP-related risk model has been found to be an independent prognostic factor for predicting survival in BCa patients. The prognosis risk score from the CCP-related risk model was combined with a number of clinical characteristics to create nomogram prognostic models, which were then verified, in order to improve prognostic accuracy. In BCa patients, the nomogram showed better predictive accuracy than T stage, N stage, and clinical stage.

GO, KEGG, and GSVA enrichment analyses were used to perform a functional enrichment analysis of the CCP-related risk model. The findings demonstrated that the CCP-related risk model is involved in multiple cellular immunological activities, especially those linked to leukocytes, and impacts a broad spectrum of cell cycle-related processes. Natural killer cells and cytotoxic T cells are examples of immune surveillance cells that are crucial in identifying and destroying aberrant cells, including those that show dysregulated cell cycle progression [49].Moreover, immune cell activation and proliferation include closely controlled cell cycle activities, which include fast cell division and the production of immunological responses. Immune dysfunction may arise from any malfunction in this complex process [50].

Certain elements of the cell cycle apparatus, such as regulators and checkpoint proteins, may be targets for immunotherapy. For BCa patients, immune checkpoint inhibitors show good treatment outcomes [51, 52]. Subsequent analysis of immune infiltration patterns showed that the high-risk group had reduced infiltration of regulatory T cells and activated dendritic cells, but increased infiltration of M2 macrophages, neutrophils, and activated CD4 + memory T cells. Furthermore, the high-risk group had considerably greater enrichment scores for immunological pathways when compared to the low-risk group, with the exception of the type 2 interferon response pathway. Additionally, these risk genes were positively or negatively correlated with immune checkpoint genes. Our findings imply that CCP-related gene markers could be useful targets for immune treatment since they could contribute to an immunologically active state. Unchecked proliferation and disturbed cell cycle control are hallmarks of cancer cells. Through the direct targeting of cyclins within tumor cells or the indirect induction of an immune response against them, immunotherapy can take advantage of these abnormalities [53, 54].

The abnormal cell cycle progression can be targeted for therapeutic intervention and modulates immune responses against tumors or pathogens. Understanding how these systems interact is essential to creating cancer and other disease-fighting treatments. There are a few restrictions on this study, though. First of all, clinical trials are required for validation because to the retrospective nature of the data acquired from public sources. Second, there was insufficient clarity provided by the basic molecular pathways underpinning the CCP-related risk model in the progression of BCa, which calls for more investigation in subsequent research.

## Conclusions

Building on the results from earlier research, this study showed that the CCP-related risk model was a significant risk factor for both BCa diagnosis and prognosis. The CCP-related risk nomogram has been validated, which emphasizes its potential reliability as a prediction model for BCa and the need for more investigation through clinical translational research.

### Supplementary Information

Supplementary materials 1 (DOCX 3251 KB)

## Data Availability

The datasets used and/or analysed during the current study are available from the corresponding author on reasonable request.
