# Supplementary material for: Prognostic significance and immune landscape of a cell cycle progression-related risk model in bladder cancer
Source: Discov Oncol. 2024 May 12;15:160. doi: 10.1007/s12672-024-01008-x (PMC11089032; doi:10.1007/s12672-024-01008-x)
Supplement: Supplementary file 1 — Supplementary materials 1 (DOCX 3251 KB) [file 12672_2024_1008_MOESM1_ESM.docx]

**Supplementary Material**

**Supplementary Figures and Tables**

**
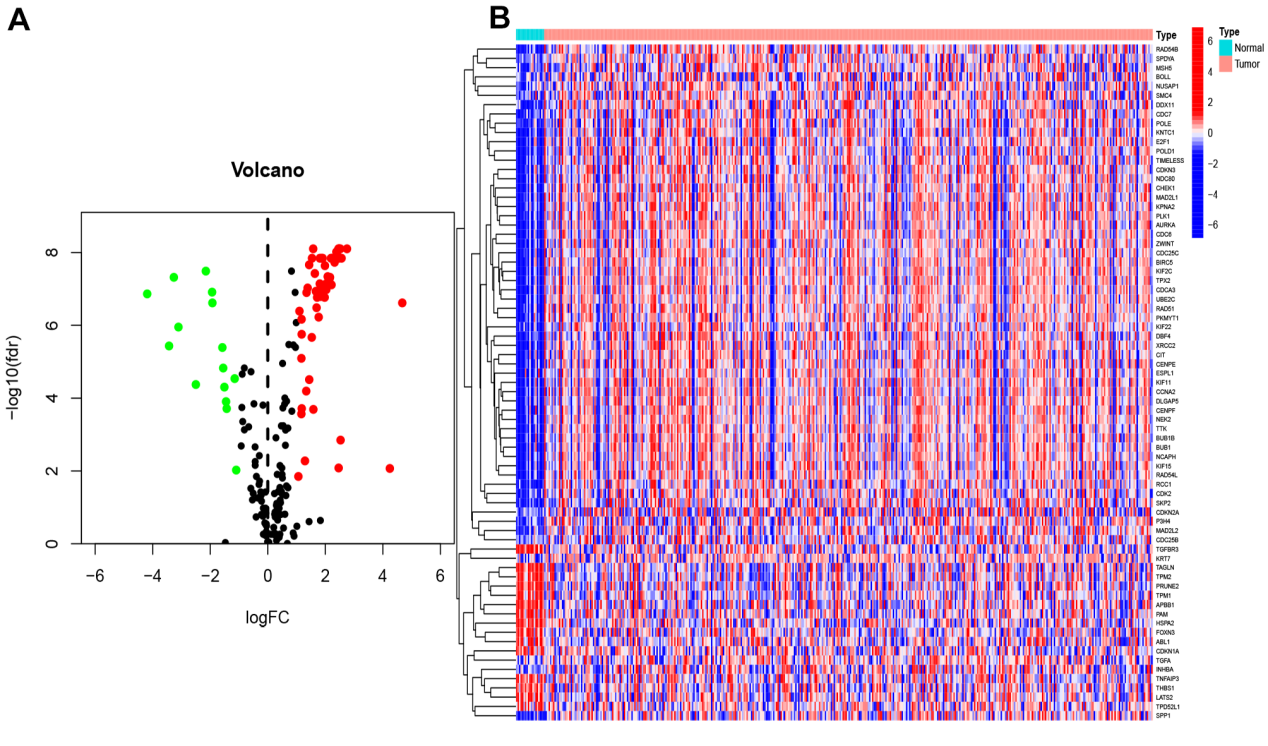
Figure S1. Gene Set Enrichment Analysis (GSEA) of ORC6 across cancers. A** Volcano map of differential expressed CCP-related genes in the TCGA-BLCA cohort. **B** Heatmap of differential expressed CCP-related genes in the TCGA-BLCA cohort.


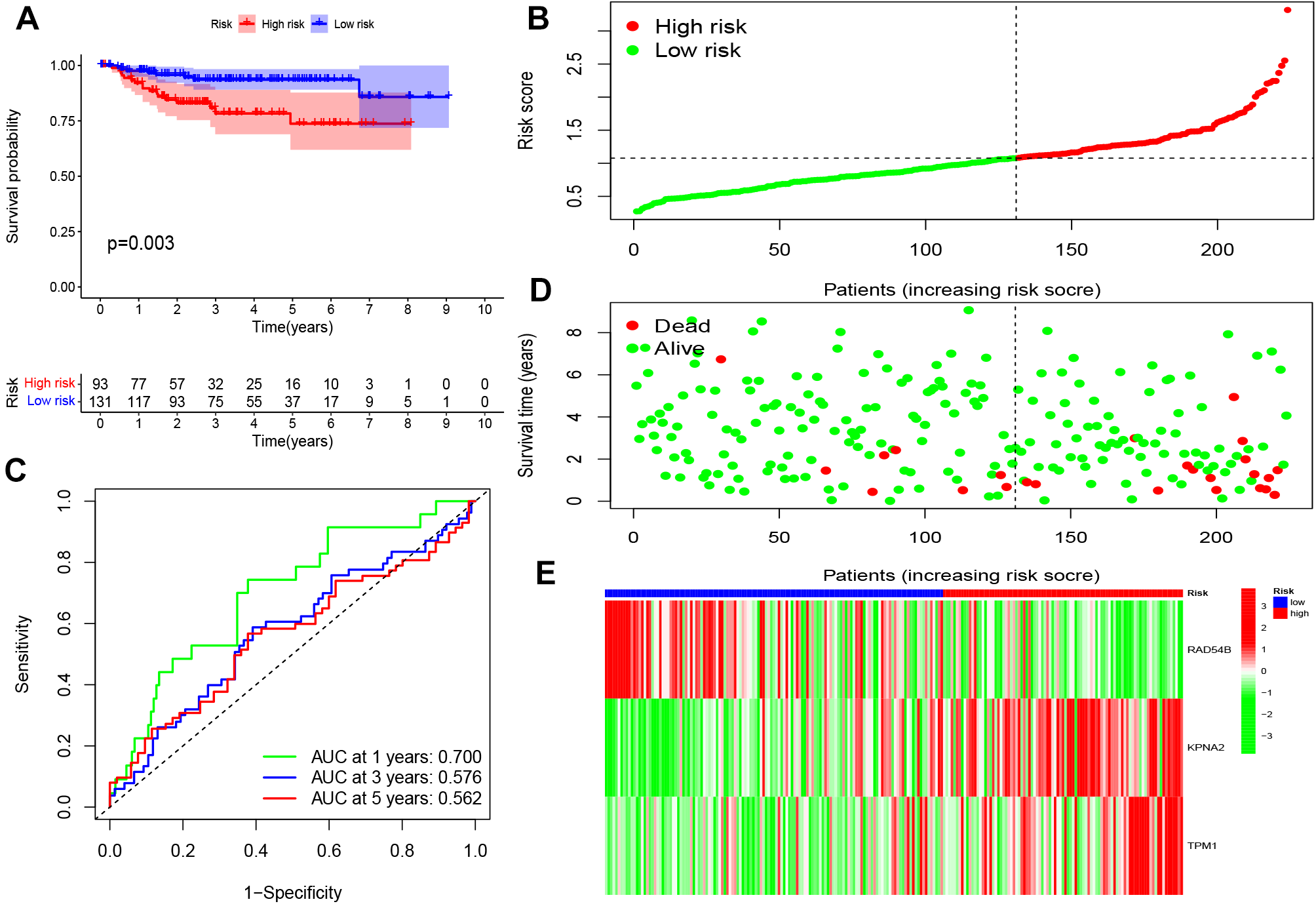


**Figure S2. Validation of the performance of the risk model using GEO dataset.** **A** Kaplan-Meier curves of OS in BCa from GEO database. **B** Distribution of risk scores among BCa patients. **C** Time-dependent ROC curves and AUCs of BCa patients for 1-year, 3-year, and 5-year survival predictions. **D** Distribution of patients who survived and died for each risk score among BCa patients. **E** Heatmap of three CCP-related genes among BCa patients.


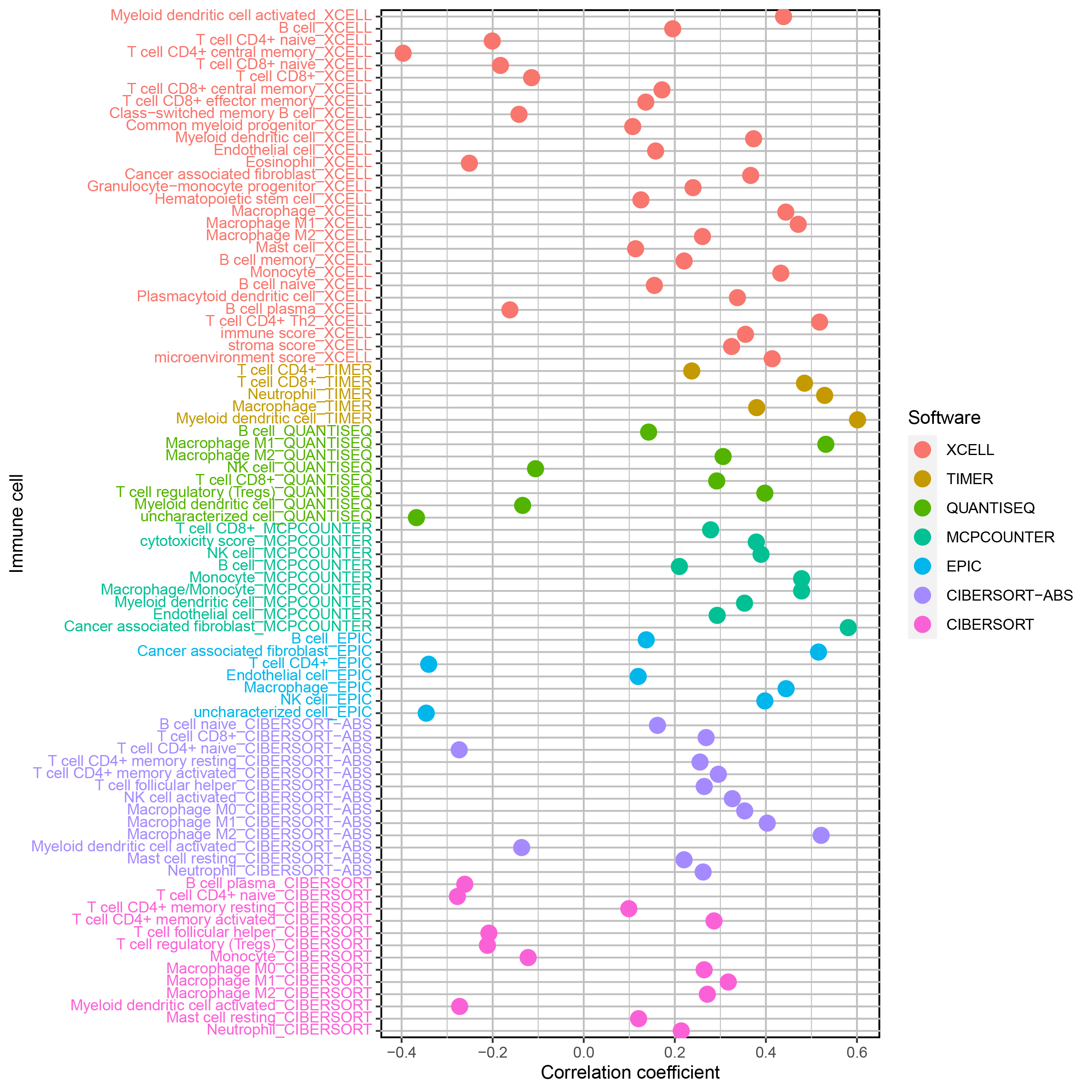


**Figure S3. Association between immune cell infiltration and different risk groups.**
